# Anti-Inflammatory Drug Design Using a Molecular Hybridization Approach

**DOI:** 10.3390/ph4111450

**Published:** 2011-10-27

**Authors:** Priscila Longhin Bosquesi, Thais Regina Ferreira Melo, Ednir Oliveira Vizioli, Jean Leandro dos Santos, Man Chin Chung

**Affiliations:** Lapdesf, Laboratory of Drug Design, Department of Drugs and Medicines, School of Pharmaceutical Sciences, University of São Paulo State (UNESP), Rodovia Araraquara-Jaú, km 1, Araraquara, SP, Cep. 14.802-901, Brazil; E-Mails: bosquesi@fcfar.unesp.br (P.L.B.); santosjl@fcfar.unesp.br (J.L.S.); ednirvizioli@yahoo.com.br (E.O.V.)

**Keywords:** anti-inflammatory, hybridization, multiple-ligands, mutual prodrug

## Abstract

The design of new drugs with better physiochemical properties, adequate absorption, distribution, metabolism, and excretion, effective pharmacologic potency and lacking toxicity remains is a challenge. Inflammation is the initial trigger of several different diseases, such as Alzheimer's disease, asthma, atherosclerosis, colitis, rheumatoid arthritis, depression, cancer; and disorders such as obesity and sexual dysfunction. Although inflammation is not the direct cause of these disorders, inflammatory processes often increase related pain and suffering. New anti-inflammatory drugs developed using molecular hybridization techniques to obtain multiple-ligand drugs can act at one or multiple targets, allowing for synergic action and minimizing toxicity. This work is a review of new anti-inflammatory drugs developed using the molecular modification approach.

## 1. Introduction

Molecular hybridization is a molecular modification approach to obtain multiple-ligands/compounds with pharmacokinetic advantages over concomitant administration of two different drugs [1]. The term “multiple ligands”, proposed by Morphy *et al*., applies to drugs that recognize more than one receptor. The advantages of multiple ligands are their ability: (1) to activate different targets by a single molecule, thereby increasing therapeutic efficacy and (2) to change the bioavailability profile in the cell and be effectively eliminated after exerting their effects [2].

**Figure 1.**
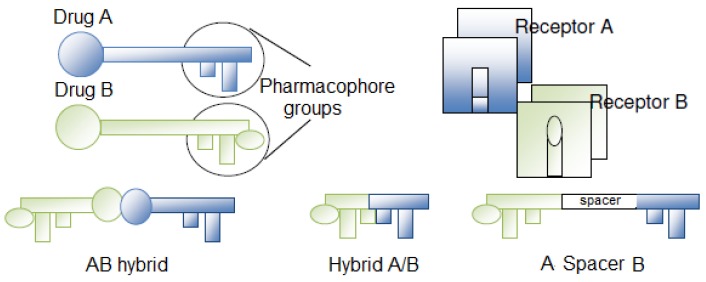
Hybridization approach. The A/B hybrid is obtained by linking the two drugs with or without a spacer subunit [3].

The hybridization process is closely related to the strategy of obtaining a mutual prodrug, with the main difference being that the prodrug action is dependent on its *in vivo* cleavage while hybrid compounds can also act “*per se*” at their specific receptors or targets (Figure 1). Hybrid compounds can be constructed by linking pharmacophore subunits directly or with spacer agents. The simple association of two distinct active principles can also be considered a hybrid compound [3].

**Figure 2.**
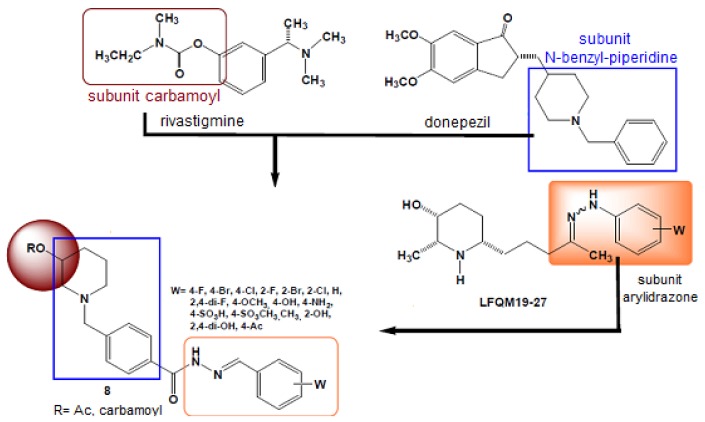
Construction of a series of hybrid NSAIDs that can interfere with the progress of the Alzheimer's disease by inhibiting the activity of acetylcholinesterase [4].

Hybridization techniques (of new entities or mutual prodrugs) to develop new anti-inflammatory drugs have been used by several researchers to obtain new compounds with better actions than those of classical nonsteroidal anti-inflammatory drugs (NSAIDs). For example, Rocha and Viegas-Jr reported using this strategy to obtain anti-inflammatory compounds useful in treating chronic degenerative diseases, such as Alzheimer's disease. The authors designed new molecules by linking together three different subunits: the carbamoyl from rivastigmine, the *N*-benzylpiperidine subunit of donezepil, and the arylhydrazone subunit of the anti-inflammatory compound LFQM 19-27 (Figure 2) [4].

New anti-inflammatory compounds were designed by the molecular hybridization of thalidomide. This drug was introduced in the pharmaceutical market in 1956 by the German Pharmaceutical Industry Chemie Grunenthal, which explored the sedative activity of the drug. In 1958, the use of thalidomide was expanded to combat nausea during pregnancy [5,6]. A few years later, however, the drug was withdrawn from the market due to its teratogenic effects [5]. In 1965, an Israeli dermatologist used thalidomide to treat his leprosy patients suffering from insomnia. To his surprise, he found that in addition to its hypnotic effects, the drug also alleviated the wounds (erythema nodosum) caused by *Mycobacterium leprae* [6-10].

The beneficial effects of thalidomide can be attributed to its anti-inflammatory, immunomodulatory, and angiogenic activities [11]. Although the mechanism of action is not yet fully understood, it is known that thalidomide inhibits chemotaxis of lymphocytes and neutrophils, and decreases the levels of cytokines such as tumor necrosis factor (TNF)-α and interferon (IFN)-γ.

In addition, thalidomide is involved in the regulation of T lymphocytes (TH1 and TH2), increases the production of TH2 and cytokines such as interleukin (IL)-4 and IL-5, and inhibits the production of inflammatory lymphocytes (TH1) and the cytokine IFN-γ in peripheral blood cells stimulated by antigens and mitogens [5,11]. In July 1998, the Food and Drug Administration approved the use of thalidomide for the treatment of erythema nodosum lepromatous [12].

**Figure 3.**
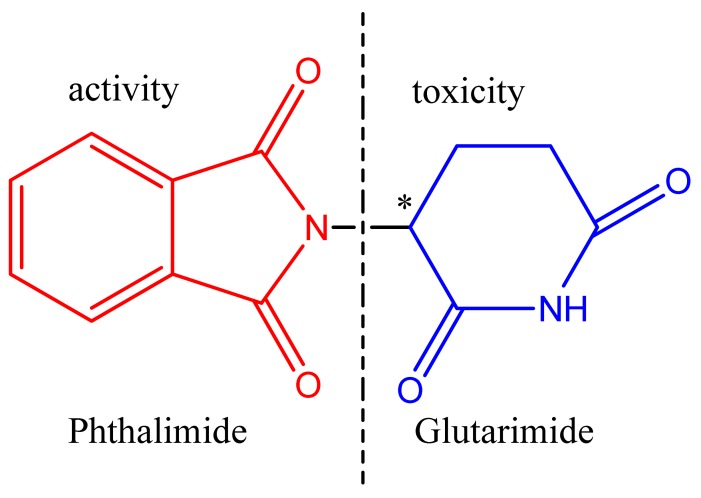
Pharmacophore and toxicophore group of thalidomide.

Several research groups have now developed thalidomide analogues for the treatment of chronic inflammation using hybridization techniques to improve the pharmacodynamic and pharmacokinetic properties, and thereby reducing the teratogenic effects. The design of new thalidomide analogues devoid of teratogenicity resulted from studies demonstrating that the teratogenic effects are due to the toxicophore glutarimide subunit (Figure 3) [9].

**Figure 4.**
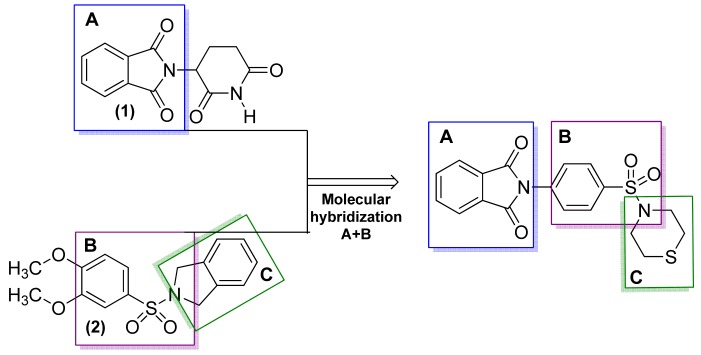
Hybrid of TNFα and phosphodiesterase–inhibiting compound with anti-asthma activity [20].

Patients with chronic inflammatory diseases have elevated levels of the pro-inflammatory cytokine TNF-α, which triggers a series of detrimental changes that promote the development of inflammatory, immunopathologic, and autoimmune diseases [13-19]. One such disease, asthma, affects the airways involving many cells and cellular elements, particularly mast cells, eosinophils, T lymphocytes, macrophages, and neutrophils of epithelial cells. The inflammatory process also causes an associated increase in the existing exacerbated bronchial response to a variety of stimuli. Based on this knowledge, Lima and coworkers obtained a series of new *N*-substituted phthalimide derivatives, structurally designed as hybrids of thalidomide (**1**) and aryl sulfonamides (**2**), to selectively inhibit phosphodiesterase, which is involved in the asthma process (Figure 4) [20].

**Figure 5.**
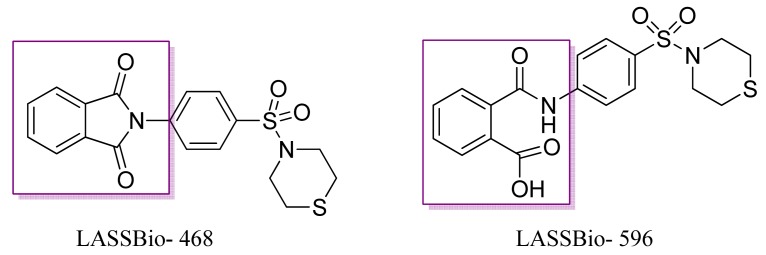
Prototype anti-asthmatic LASSBio hybride-468 and its active metabolite LASSBio-596.

These authors showed that the best compound, LASSBio 468 (Figure 5), inhibited the neutrophil infiltration induced by lipopolysaccharides (LPS) with an ED_50_ of 2.5 mg kg^−1^ as well as the increased TNF-α in the bronchoalveolar lavage fluid from mice treated with LPS. The preliminary structure activity relationship of this compound revealed the importance of the sulfonyl group, the nature of the *N*-terminal piperazine ring, and the role of the phthalimide ring in the anti-inflammatory activity of this compound (468) [20]. LASSBio-596 is the metabolic compound of LASSBio-468, with activity similar to that of the parent drug.

**Figure 6.**
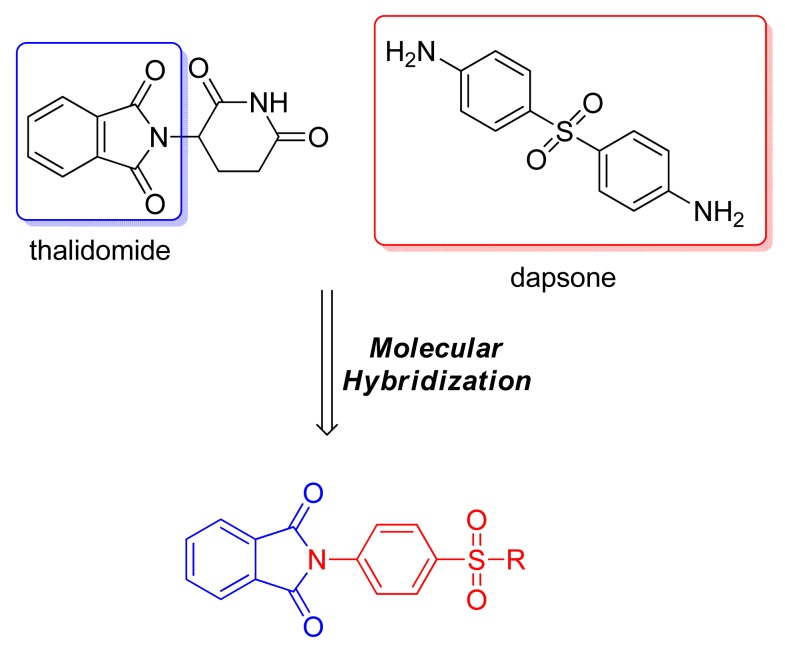
Hybrid of thalidomide anti-inflammatory moiety and dapsone effective against *Mycobacterium tuberculosis*, with anti-inflammatory and analgesic activity.

Using the same concept of the phthalimide moiety as a TNF-α inhibitor, Santos and coworkers used molecular hybridization to develop a series of derivatives of thalidomide and dapsone to obtain new anti-inflammatory compounds to treat erythema nodosum lepromatous (Figure 6). All the compounds demonstrated analgesic/anti-inflammatory activity and reduced *in vitro* TNF-α levels. Furthermore, the compounds reduced the number of bacillus/paw in nude mice 12 months after inoculation of 1.0 × 10^4^ bacilli/mL in comparison with controls (unpublished results). Curiously, when the same compounds were evaluated against *Mycobacterium tuberculosis*, three of them had minimum inhibitory concentrations of 3.9, 5.0, and 7.8 (μg/mL). These compounds demonstrated an excellent selectivity ratio, decreasing the number of *M. tuberculosis* without macrophage toxicity [21].

Santos and coworkers also used a phthalimide moiety to construct new hybrid derivative drug candidates useful for treating the symptoms of sickle cell disease. This disease is characterized by a point mutation that changes glutamic acid (Glu6) to valine (Val6) in the β chain of hemoglobin. In the deoxy state, this modification leads to polymerizations that modify the cytoskeletal structure to form a sickle morphology. Patients with sickle cell disease present with a well-known chronic inflammatory condition. The only drug approved by the Food and Drug Administration to treat sickle cell disease is the antineoplasic hydroxyurea, which controversially increases the expression of pro-inflammatory cytokines.

**Figure 7.**
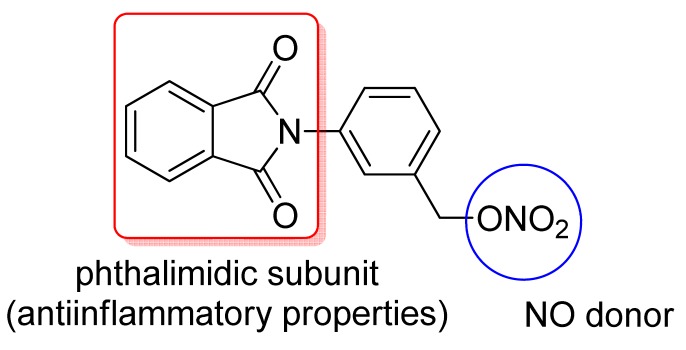
Hybrid of TNF-α inhibitor/NO donor for the treatment of sickle cell disease symptoms.

Based on the inflammatory aspects of sickle cell disease, Santos and coworkers developed new compounds from thalidomide and hydroxyurea (Figure 7). The synthesized compounds had higher analgesic and anti-inflammatory activities than controls and reduced the TNF-α levels of transgenic sickle animals. Furthermore, the important property of hydroxyurea to induce gammaglobulin expression was maintained and, surprisingly, the potency of the compounds was increased compared with that of hydroxyurea. Thus, these results showed the successful application of this molecular modification tool to improve the pharmacodynamic properties of new drug candidates to treat sickle cell disease [22].

**Figure 8.**
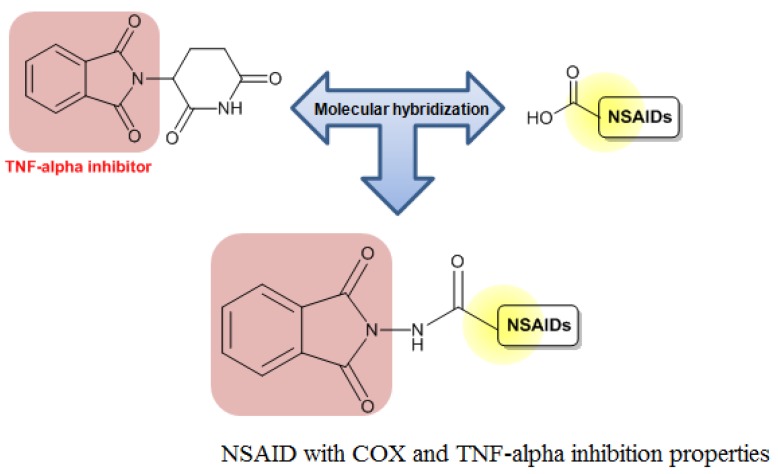
General structures of NSAID hybrids with COX and TNF- α inhibiton properties. NSAID = salicylic acid, diclofenac, naproxen, ibuprofen, ketoprofen.

In addition, Santos and coworkers obtained a series of hybrid derivatives of classical NSAIDs with a phthalimide group (Figure 8). These hybrid compounds may act synergistically, inhibiting cyclooxygenase (COX)-2 and TNF-α with potential activity against chronic inflammation [23]. Indeed, Vizioli (2009) showed that these hybrid NSAIDs were effective against acute anti-inflammatory and ulcerative colitis and showed no gastrotoxicity [3].

Jung and coworkers obtained a prodrug of mesalasine and taurine to selectively target the colon. Extensive mechanistic studies, however, showed that chlorinated taurine (Tau-Cl) has additive anti-inflammatory activity by inhibiting IL-1b mediated nuclear factor-κB activation [24,25].

**Figure 9.**
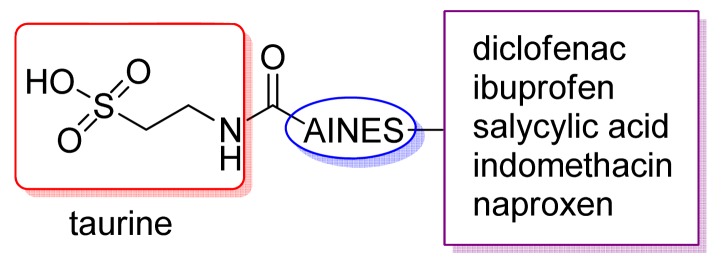
Mutual prodrugs of NSAIDs and taurine with COX and IL-1 inhibition activity without gastrotoxicity.

Vizioli reported the synthesis and biologic activity of mutual prodrugs of classical NSAIDs and taurine (Figure 9). These compounds showed no gastric toxicity and abolished ulcerative colitis induced by acetic acid in rats, decreasing mortality in comparison with NSAIDs [26,27].

Excessive production of reactive oxygen species is associated with inflammation and leads to the condition of oxidative stress, which can contribute to the high mortality rates associated with several diseases [28].

Natural and synthetic antioxidants possess anti-inflammatory properties [29,30]. Lipoic acid (1,2-dithiolane-3-valeric acid) is a naturally occurring compound present in all kinds of prokaryotic and eukaryotic cells and synthesized by animals. It exists as both the reduced dithiol form (dihydrolipoic acid) and the oxidized disulfide form [31,32] and has been described as a universal antioxidant that reacts with RO_2_^•^, ascorbyl radicals HO^•^ and NO^•^, and tocopherol radicals O_2_^•−^ and hypochlorous acid [33-35].

**Figure 10.**
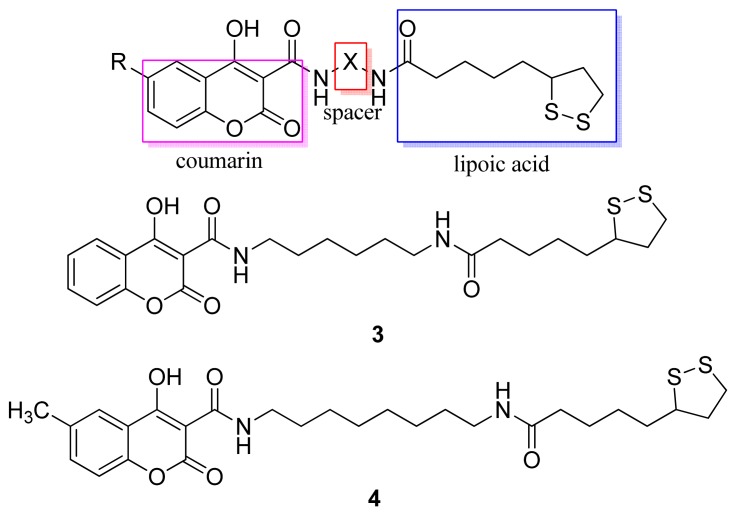
Hybrid of coumarin/lipoic acid.

Melagraki and coworkers developed hybrid compounds of lipoic acid and coumarins [36]. Coumarins exhibit anti-inflammatory and antioxidant activities [37,38]. The inhibition of inflammation ranged from 41% to 73%, while the reference drug indomethacin showed 47% inhibition at an equivalent dose. All compounds showed activity, but conjugates **3** and **4** (Figure 10) were more potent [36].

**Figure 11.**
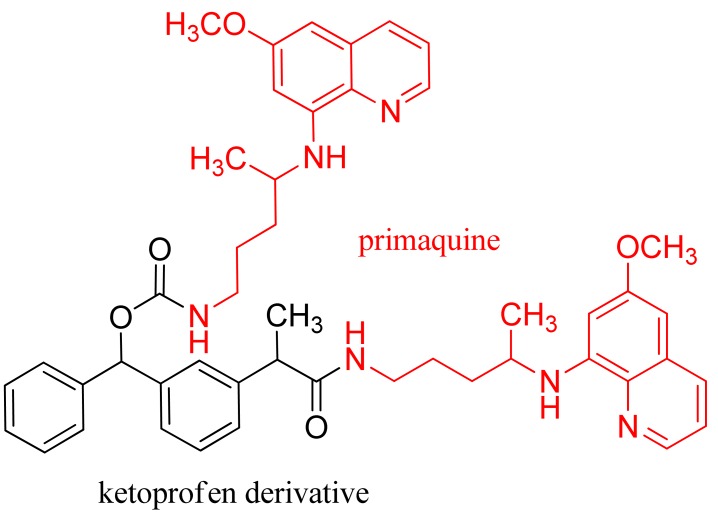
Hybrid of primaquine-ketoprofen (compound **5**).

The increase in reactive oxygen species is associated with death caused by malaria due to erythrocyte hemolysis. Based on this concept, Raji and coworkers obtained a NSAID (ibuprofen, ketoprofen, fenoprofen, ketoprofen hydroxy) and methylene analogue (diclofenac or indomethacin) hybrid of primaquine (PQ) to decrease the inflammatory activity of reactive oxygen species in malaria. Compound **5** comprises one reduced ketoprofen moiety and two PQ units (one bound by an amide and the other by the carbamate bond) (Figure 11). All the tested PQ-NSAID conjugates showed antioxidant activity and moderate antiradical activity, with an *EC*_50_ between 269.5 ± 10.7 and 379.3 ± 59.1 mg L^−1^. Based on the *β*-carotene-linoleic acid assay, the PQ-ketoprofen hybrid (compound **5**) possessed the strongest antioxidant activity. The most active conjugate, according both to the antioxidant activity relative to water control value (69.4 ± 0.9%) or the absolute changes in absorbance at *t* = 60 (58.4 ± 3.1%) and *t* = 120 min (59.3 ± 1.8%), was compound **5** with two PQ units [39].

Nitric oxide (NO), which is naturally generated from L-arginine by NO synthase, is a key signaling molecule involved in the regulation of many physiologic processes, including vascular relaxation, neurotransmission, and immune system events.

Most recent studies of hybrid anti-inflammatory agents report using NO donors as a moiety to improve activity in treating several diseases, including atherosclerosis, which is a disease related to endothelial dysfunction resulting from an increase in plasma lipids, peroxidation of low-density lipoproteins, and impaired endothelial-derived relaxing factor (NO, NO^•^)-mediated bioactions. The oxidative stress of low-density lipoproteins leads to the formation of foam cells, the precursors of atherosclerotic plaques. In an atherosclerotic blood vessel, NO^•^ bioactions are impaired by a number of processes, including a possible decrease in NO^•^ production, an increase in NO^•^ inactivation, and a decrease in the responsiveness of the target cells to NO^•^.

**Figure 12.**
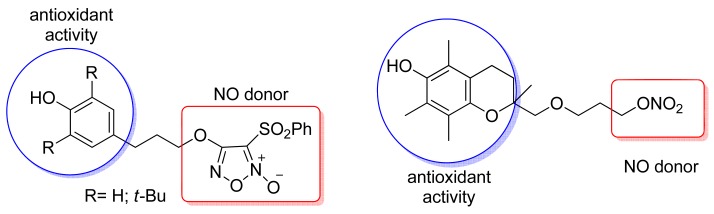
Hybrid anti-inflammatory antioxidant and NO donor.

Cena and coworkers obtained a series of hybrids of ascorbic acid (antioxidant derivatives)/NO donor (furoxans, nitrate), as shown in Figure 12. All compounds inhibited ferrous salt/ascorbate-induced lipid peroxidation of membrane lipids of rat hepatocytes and showed potent *in vitro* vasodilation activity. These hybrids promoted dose-dependent dilation of rat aorta strips pre-contracted with phenylephrine [40].

**Figure 13.**
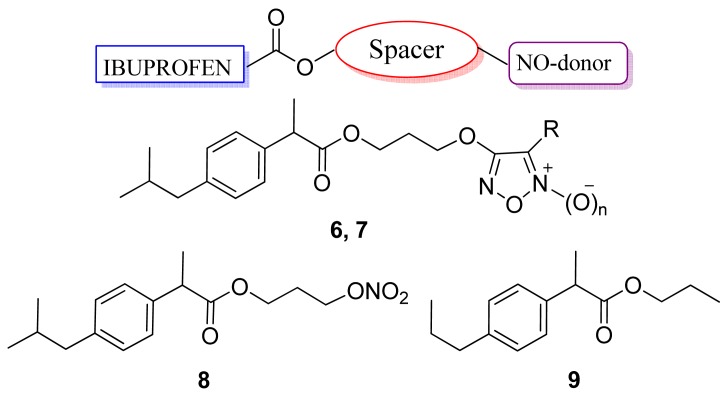
Hybrid ibuprofen/NO donor compounds (**6** n = 1, R = PhSO_2_; **6a** n = 0, R = PhSO_2_; **7** n = 1, R = PhS; **7a** n = 0, R = PhS.

Several researchers have synthesized derivatives of NSAIDs with NO donor moieties. In 2001, a series of NSAIDs obtained by linking ibuprofen to selected furoxan moieties and related furazans were synthesized and tested for their anti-inflammatory, antiplatelet, and anti-ulcerogenic properties (Figure 13). All the derivatives showed anti-inflammatory activities comparable to that of ibuprofen and, unlike ibuprofen, they showed reduced acute gastrotoxicity [41].

Ibuprofen propyl ester **9** significantly reduced edema after 4 and 6 hours (37.6% and 49.6%, respectively). All the other products of the series also showed significant anti-inflammatory activity, with comparable efficacy, less irritation, and less platelet aggregation than ibuprofen. With regard to platelet aggregation, the NO-donor **6** was more potent than ibuprofen, while compounds **7** and **8** were approximately equipotent with the parent drug [41].

**Figure 14.**
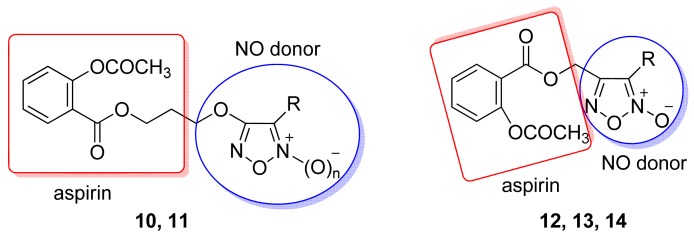
Hybrid of aspirin/NO donor (**10**, n = 1, R = PhSO_2_; **10a**, n = 0, R = PhSO_2_; **11**, n = 1, R = Ph; **11a**, n = 0, R = Ph; **12**, R = CH_3_; **13**, R = CONH_2_, **14**, R = CN) [42].

Cena and coworkers reported in 2003 a series of hybrids of aspirin/NO donors linked to furoxan moieties, with different NO-releasing abilities (Figure 14) [42]. All the compounds showed anti-inflammatory activity in a carrageenan rat paw edema model. Derivatives **11**, **13**, and **14** showed significant anti-inflammatory and antiplatelet activities, without gastrotoxicity. The most potent antiplatelet compounds of the series were the benzenesulfonyl and cyano-substituted furoxans **10** and **14**, respectively [42]. Turnbull and coworkers tested the TNF-α inhibition properties of these aspirin/NO donor hybrid compounds. Compound **14** (R = CN) had a significant inhibitory effect on TNF-α release in human monocyte-derived macrophages treated with LPS (36 ± 10% of LPS control, P < 0.01; n = 5–10). The effect was equivalent in magnitude to that of dexamethasone, but was not shared by DEA/NO (2-(*N,N*-diethylamino)-diazenolate-2-oxide), furazan, aspirin, or NCX4016 (3-(nitroxymethyl)phenyl 2-(acetoxy)benzoate).

None of the treatments studied caused significant cell death compared with untreated macrophages and monocytes. Levels of lactate dehydrogenase released following the treatments were comparable with those released from untreated control samples (∼0.5 × 105 cells/treatment) [43].

**Figure 15.**
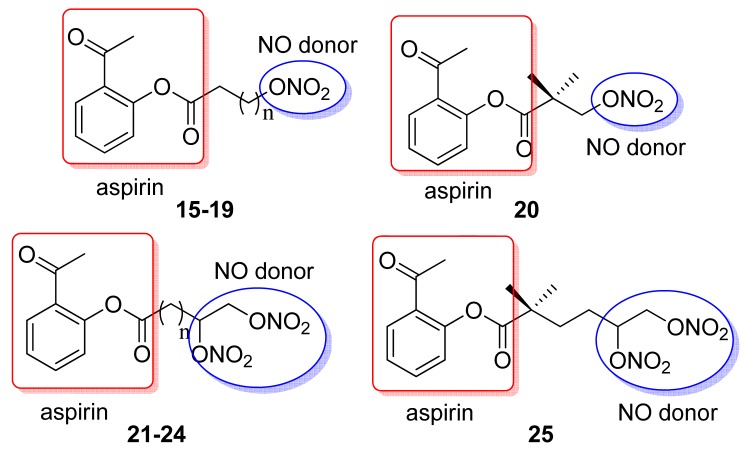
Hybrid aspirin/NO derivatives (**15** n = 1; **16** n = 2; **17** n = 3; **18** n = 4; **19** n = 5; **21** n = 1; **22** n = 2; **23** n = 3; **24** n = 4) [44].

Based on the same concept, Gasco and coworkers designed a class of NO-donor aspirin-like drugs. These hybrid compounds were derived from aspirin by linking acyl moieties possessing NO-donor *O*-nitro groups to the −OH function of salicylic acid (Figure 15) [44].

Oral administration of mono *O*-nitro derivatives **15-20** and di (*O*-nitro) derivatives **21-25** to rats at a dose equimolar with aspirin (120 mg/Kg i.g.) showed less gastric damage after 3 h compared with aspirin. These derivatives also inhibit platelet aggregation induced by collagen in human platelet-rich plasma. Compounds **15** and **21** were the best antiplatelet compounds and due to the presence of *O*-nitro NO-donor moieties, all the compounds promoted relaxation in rat aorta strips pre-contracted with phenylephrine [44].

In 2009, a series of nitrooxyacyloxy methyl esters of aspirin were synthesized and evaluated as new NO-donor/aspirin hybrids by Lazzarato and coworkers using nitrates as the NO donor moieties. All the compounds showed *in vitro* vasodilator activity, but no antiplatelet activity. The best results were obtained using aromatic nitrooxyacyloyl moieties.

**Figure 16.**
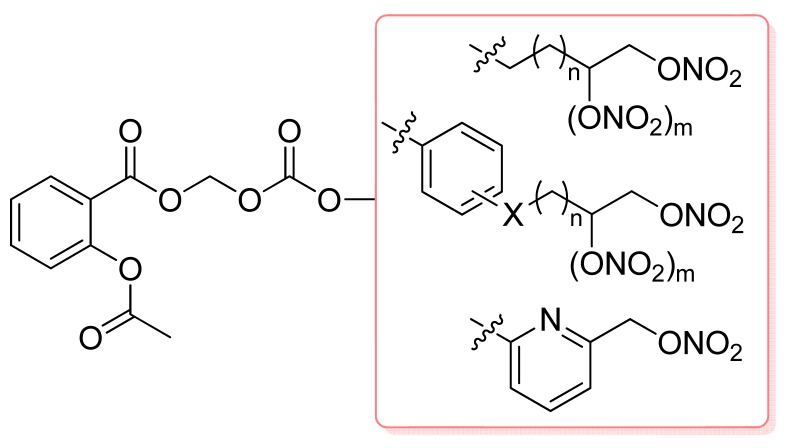
Nitrooxyacyloxy methyl esters of aspirin derivatives (n = 0–3; m = 0–1; X = O, CH_2_, S, SO and SO_2_).

In the case of the benzoyl derivatives, the most active compounds presented NO-donor chains at the *p*- or at *m*-position (Figure 16). All the compounds, including the nitrooxy-substituted acid metabolites displayed *in vitro* vasodilator activity. The aromatic derivative series showed higher activity than the aliphatic derivative series [45].

**Figure 17.**
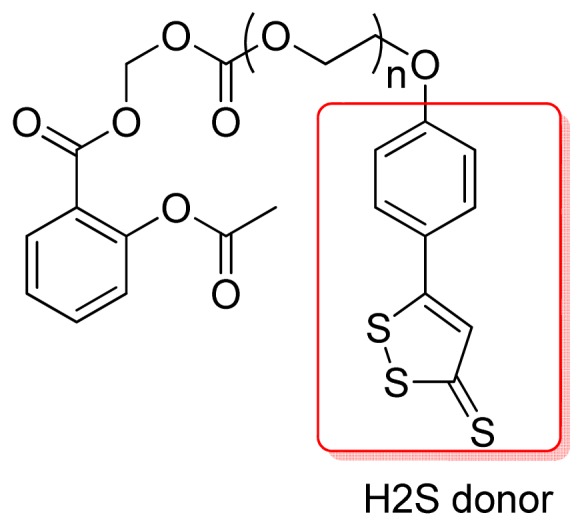
Hybrid of aspirin with H_2_S donor properties (n = 0; n = 1).

In 2011, the same authors obtained an aspirin hybrid with H_2_S donor properties (Figure 17) and all compounds were stable in acid and physiologic pH and promoted fast aspirin release when incubated in human serum. The compounds inhibited collagen-induced platelet aggregation in human plasma. This effect was observed only with hybrid compounds while the control NO-donor and H_2_S-donor moieties did not show anti-platelet activity. These new compounds produce the expected NO-dependent and H_2_S-dependent vasodilator activities, respectively, and the authors suggested the use of these compounds as safer derivatives of aspirin for antithrombotic and anti-inflammatory therapies [46].

In addition, NSAIDs such as aspirin have shown promising effects in colorectal cancer clinical trials. Exploration of the use of NO with NSAIDs (NO/NSAIDs) in chemotherapy represented a logical progression, because NO/NSAIDs were originally designed to deliver the biologic activity of NO to reduce the gastrointestinal damage caused by NSAIDs [47].

**Figure 18.**
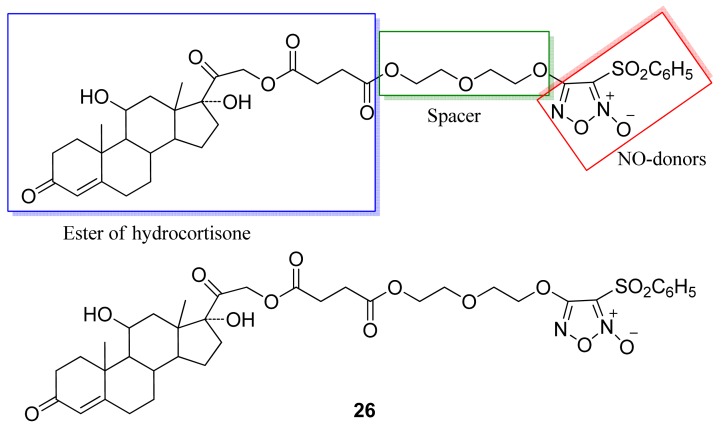
Hybrid of glucocorticoid/NO donor.

Glucocorticoids have been widely used since the 1950s for their potent anti-inflammatory and immunosuppressive actions [48]. Fang and coworkers obtained a series of hybrid compound derivatives of glucocorticoids with NO donors (Figure 18) [49].

The results of Fang and coworkers showed that compound **26** was effective against acute and chronic inflammation (paw edema and rheumatoid arthritis model in rats). The most important effect in chronic inflammation is the higher activity observed than when using hydrocortisone alone in the later phase of the disease (days 21 and 24) [49].

**Figure 19.**
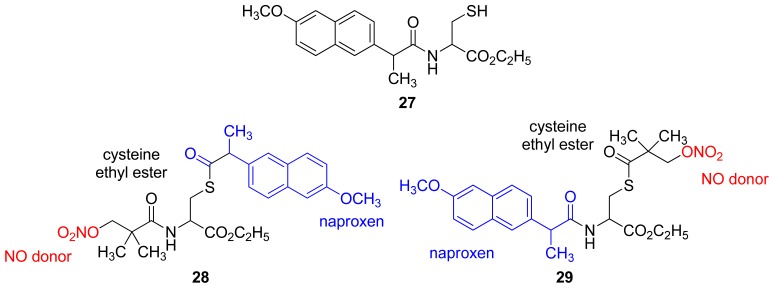
Triple hybrid of naproxen, cysteine ethyl ester, and nitrooxypivaloic acid (**28** and **29**). Hybrid intermediate of naproxen and cysteine (**27**).

Two triple-hybrid compounds containing a cysteine subunit (known to enhance the activity of organic nitrates and reduce nitrate tolerance), naproxen, and an organic nitrate (nitrooxypivaloic acid) were synthesized. Derivatives **28** and **29** (Figure 19), comprising naproxen, cysteine ethyl ester, and nitrooxypivaloic acid, showed good anti-inflammatory activity and decreased gastrointestinal tract damage. This new hybrid compound showed a reduced induction of tolerance and an attenuated decrease in blood pressure with nitrates (data not published) [50]. The authors, however, showed only the *in vitro* results.

Intermediate **27** and compounds **28** and **29** were tested for their COX inhibitory properties relative to naproxen. Naproxen inhibits COX-1 with an IC_50_ of 0.1 μM and COX-2 with an IC_50_ of 9.0 μM. Compound **27** showed no inhibition of COX-2 and weakly inhibition of COX-1 (IC_50_ = 6.0 μM). The *N*-3-nitrooxypivaoyl-*S*-(+)-2-(6-methoxy-2-naphthyl)-propanoyl-l-cysteine ethyl ester (**28**) was inactive against both COX-1 and COX-2 at a concentration range of 0.1 to 10 μM, while *N*-(+)-2-(6-methoxy-2-naphthyl)propanoyl-*S*-3-nitrooxypivaloyl-l-cysteine ethyl ester (**29**) exhibited no COX-2 inhibition activity at the same concentration range and low COX-1 inhibition activity with an IC_50_ of 5.6 μM [50].

**Figure 20.**
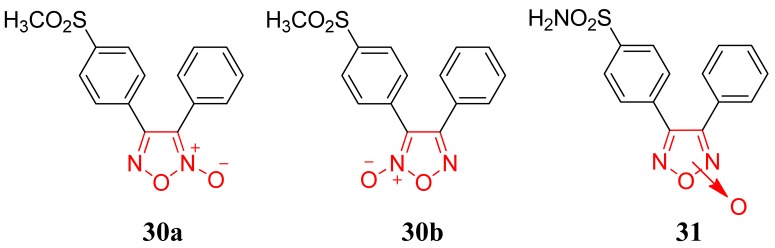
COX-2 inhibitors (black)/NO donor (red) hybrid agents.

Knaus and coworkers obtained a series of selective COX-2 inhibitor/NO donor hybrid agents (Figure 20). 3,4-Diphenyl-1,2,5-oxadiazole-2-oxides (3,4-diphenylfuroxans) and the corresponding *N*-desoxy 3,4-diphenyl-1,2,5-oxadiazoles (3,4-diphenylfurazans) analogs were evaluated and showed COX-2 selectivity. The methanesulfonyl regioisomers **30a**,**b** [COX-1 IC_50_ = 11.6 μM; COX-2 IC_50_ = 0.12 μM; COX-2 selectivity index (SI) = 97] and aminosulfonyl regioisomers **31** (COX-1 IC_50_ = 9.8 μM; COX-2 IC_50_ = 0.78 μM; COX-2 SI = 12), reference drug celecoxib (COX-1 IC_50_ = 33.1 μM; COX-2 IC_50_ = 0.07 μM; COX-2 SI = 472), were potent *in vitro* COX-2 inhibitors with a good COX-2 selectivity index.

The authors suggested that the 1,2,5-oxadiazole-2-oxide (furoxan) ring system possesses beneficial features desirable for the design of hybrid COX-2 inhibitor/NO donor anti-arthritic agents with a low ulcerogenicity profile and minimal potential to induce adverse cardiovascular events such as heart attacks and strokes [51].

**Figure 21.**
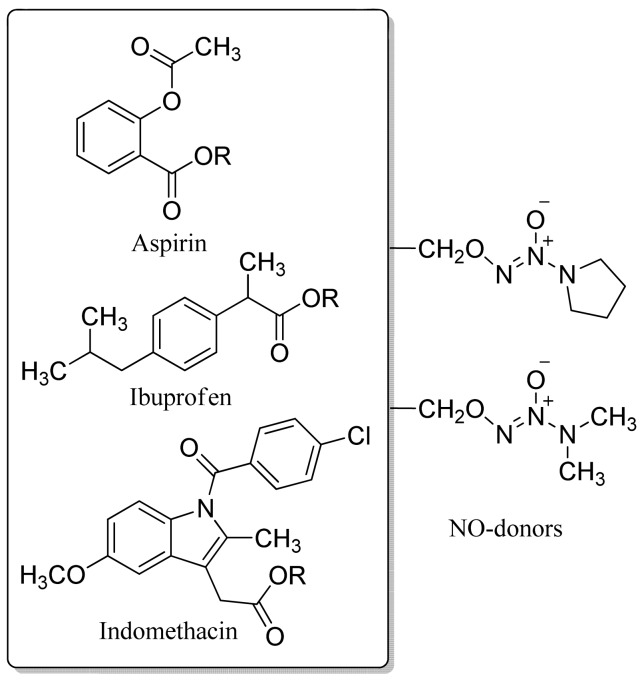
Hybrid of a classical NSAID and NO donor.

The same authors obtained a series of hybrid NO-releasing NSAIDs possessing a 1-(pyrrolidin-1-yl)diazen-1-ium-1,2-diolate or 1-(*N,N*-dimethylamino)diazen-1-ium-1,2-diolate moiety attached via a one-carbon methylene spacer to the carboxylic acid group of the classical NSAIDs aspirin, ibuprofen, and indomethacin (Figure 21) [52].

*In vitro* studies of COX showed that none of these compounds inhibited either the COX-1 or COX-2 isoenzyme at the highest test compound concentration used (100 μM). When administered orally to rats, however, the carrageenan-induced rat paw edema assay indicated ID_50_ values similar to those obtained for the reference drugs [52].

**Figure 22.**
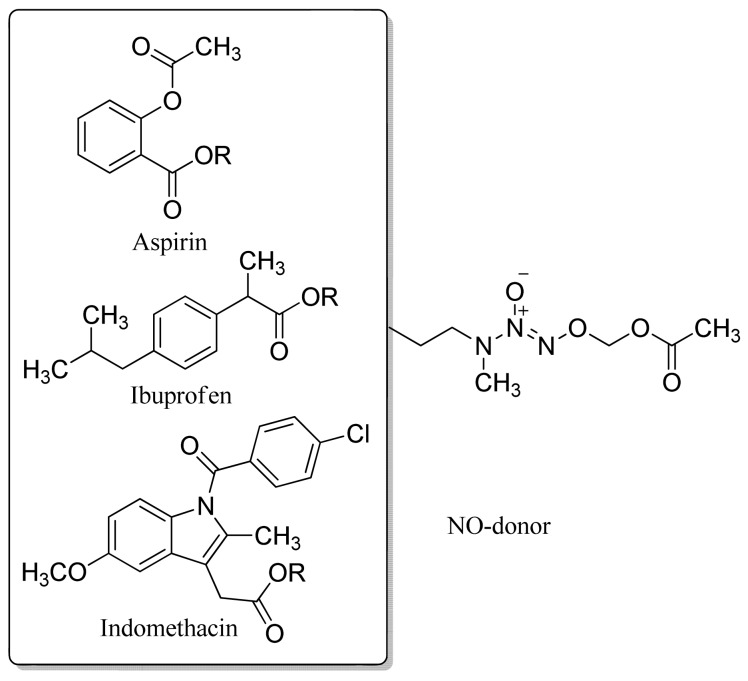
Mutual NONO/NSAID prodrug derivatives.

In 2007, Knaus and coworkers tried other NO donor groups as NSAID linkers. They obtained NO-releasing nonsteroidal anti-inflammatory mutual prodrugs possessing an *O*-2-acetoxymethyl-1-[*N*-(2-hydroxyethyl)-*N*-methylamino]diazen-1-ium-1,2-diolate moiety (NONO-NSAIDs) (Figure 22) [53].

These derivatives did not inhibit the catalytic activity of COX-1/COX-2 isozymes *in vitro,* but showed equipotent anti-inflammatory properties compared to their parent NSAID in a carrageenan-induced rat paw edema model *in vivo,* without significant gastric toxicity, when administered orally. The authors report the benefits of using mutual NONO-aspirin prodrugs for the prevention of thrombus formation and adverse cardiovascular events such as stroke and myocardial infarction [53].

**Figure 23.**
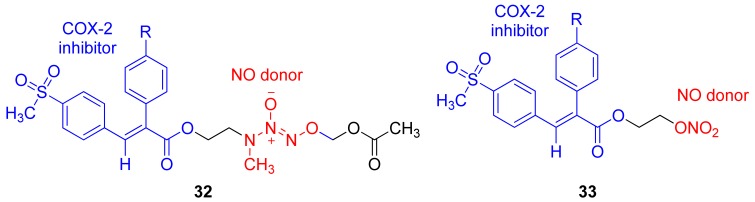
Hybrid of COX-2 and NO derivative developed by Knaus and coworkers (2008), (R = **a** = H; **b** = OCH_3_; **c** = F; **d** = Br).

In 2008, Knaus and coworkers obtained a nonselective COX hybrid of NO donor anti-inflammatory compounds wherein an *O*-2-acetoxymethyl-1-(*N*-ethyl-*N*-methylamino)diazen-1-ium-1,2-diolate (**32a-d**), or 2-nitrooxyethyl (**33a-d**) NO-donor moiety is attached directly to the carboxylic acid group of (*E*)-3-(4-methanesulfonylphenyl)-2-(phenyl)acrylic acids (Figure 23). The *O*2-acetoxymethyl-1-(*N*-ethyl-*N*-methylamino)diazen-1-ium-1,2-diolate (**32a-d**) derivatives undergo extensive ester cleavage by esterase (in rats serum), followed by a significant release of NO (∼76.2%−83.0%) [54].

**Figure 24.**
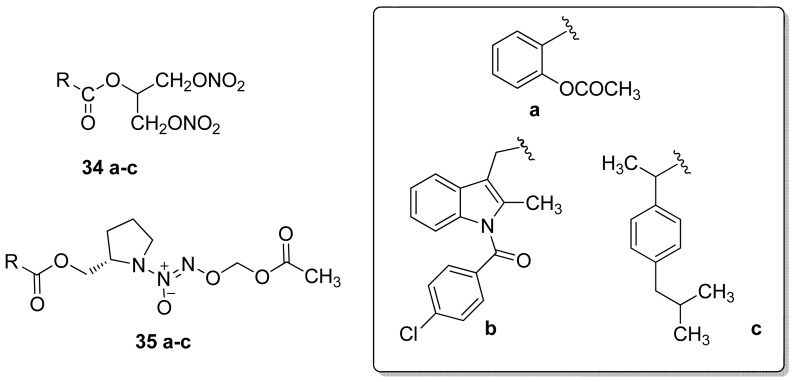
NONO-NSAID hybrid derivatives obtained by Knaus and coworkers.

In 2009, the same authors developed NONO/NSAIDs wherein a 1,3-dinitrooxy-2-propyl (**34a–c**), or *O*2-acetoxymethyl-1-[2-(methyl)pyrrolidin-1-yl]diazen-1-ium-1,2-diolate (**35a–c**) NO-donor moiety was directly linked to the carboxylic acid group of aspirin, indomethacin, or ibuprofen (Figure 24). All compounds showed COX-2 activity without COX-1 enzyme inhibition at the highest concentration used (100 μM) [55].

**Figure 25.**
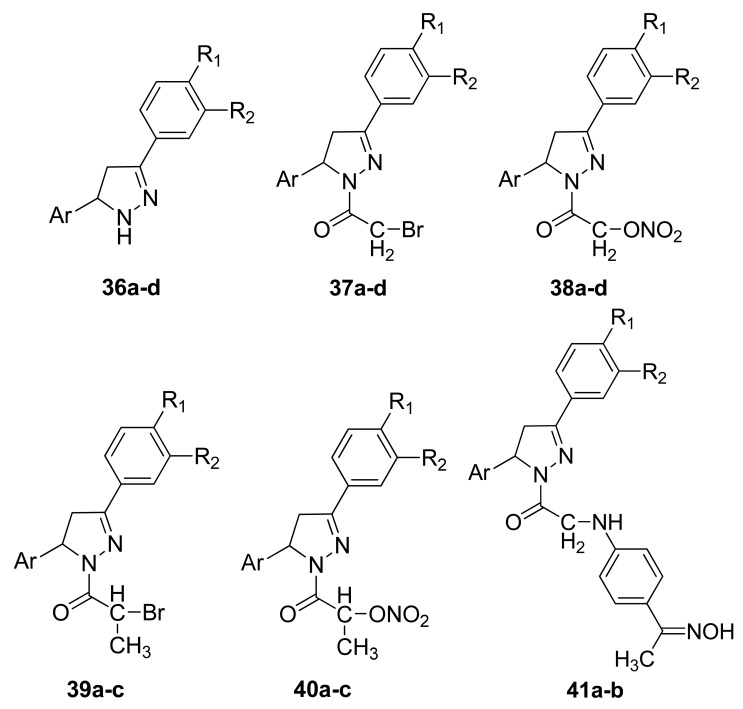
Diaryl pyrazoline hybrid derivatives (**a** R_1_ = R_2_= OCH_3_, Ar = Furyl; **b** = R_1_ = R_2_= OCH_3_, Ar = 2,4-Di-OCH_3_phenyl; **c** R_1_ = R_2_ = OCH_3_, Ar = 2,6-Di-Cl phenyl; **d** R_1_ = OCH_3_, R_2_ = H, Ar = 2,6-Di-Cl phenyl).

In the same year, Shoman and coworkers obtained a series of 3,5-diaryl-2-pyrazoline derivatives via the reaction of chalcones with hydrazine hydrate in ethanol. A group of NO-donating-2-pyrazoline derivatives was synthesized by carrying a nitrate ester group or an oxime group onto the prepared pyrazoline derivatives through different spacers (Figure 25) [56].

**Figure 26.**
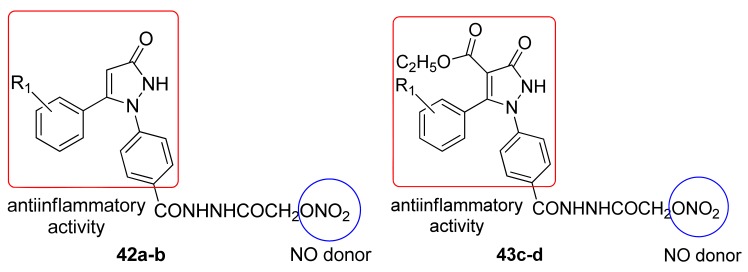
Diarylpyrazol anti-inflammatory derivatives (**42a** R = -4-NO_2_; **42b** and **43c** R = -2-OCH_3_; **43d** R = H).

Most of the prepared compounds showed significant anti-inflammatory activity at 100 mg/kg and were safer than indomethacin with respect to gastric toxicity. Incorporation of the NO-donating group into the parent pyrazoline derivatives caused a nonsignificant reduction in the anti-inflammatory activity, but there was also a marked decrease in gastric ulcerations induced by their parent drug [56]. Bhandari and coworkers obtained several substituted 1,5-diarylpyrazol-3-one derivatives **42a-d** and evaluated their analgesic, anti-inflammatory, and ulcerogenic activities, and their ability to release NO (Figure 26).

Some of these compounds exhibited analgesic and anti-inflammatory activities similar to indomethacin and all of the compounds showed analgesic activity and an absence of gastric damage compared with diclofenac [57].

**Figure 27.**
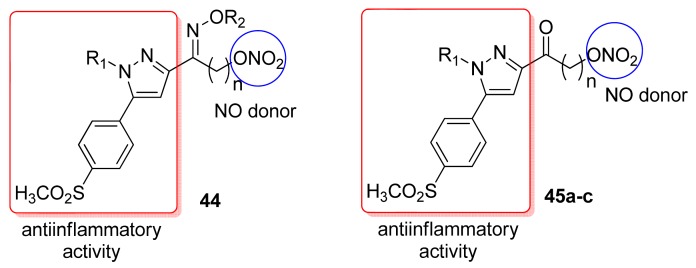
Hybrid pyrazole/NO derivatives (**44** R_1_ = cyclohexyl, R_2_ = H, n = 3; **45a** R_1_ = 4-methoxyphenyl, n = 3; **45b** R_1_ = 4-fluorophenyl, n = 3; **45c** R_1_ = cyclohexyl; n = 3).

In 2004, a series of novel pyrazoles containing a nitrate (ONO_2_) moiety functionality were synthesized by Ranatunge and coworkers. Modifications of the pyrazole moiety increased the COX-2 inhibitory potency compared to celecoxib and improved gastric tolerance (compound **44**, Figure 27). Compound **45a** proved most potent, with 78% inhibition of prostaglandin E2 formation and 43% inhibition of white cell infiltration at an oral dose of 45 μmol/kg. This compound **45a**, however, showed poor selectivity (IC_50_ for COX-1 and COX-2 was 1.5 μM and 2.5 μM, respectively). Among selective pyrazole carbonyl derivatives, only compounds **45b** and **45c** showed good anti-inflammatory activity. Both inhibited prostaglandin E2 production by 45% at 45 μmol/kg [58].

**Figure 28.**
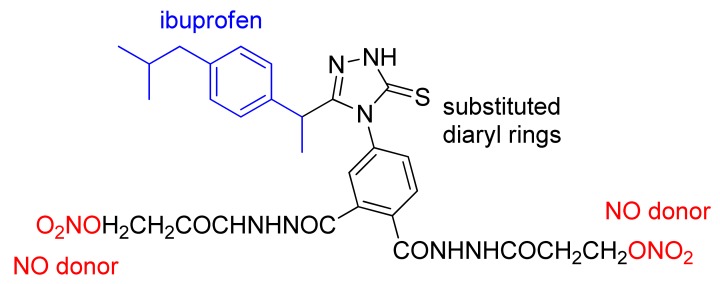
Hybrid compound with pharmacophore moiety of ibuprofen and substituted diaryl rings on a 5-membered heterocycle similar to coxibs and an NO-releasing moiety.

More recently, a series of hybrid molecules containing the pharmacophore moiety of ibuprofen and substituted diaryl rings on a 5-membered heterocycle similar to coxibs and an NO releasing moiety were obtained by Sarkate and coworkers (Figure 28). The compounds obtained showed anti-inflammatory activity similar to that of ibuprofen and decreased gastrotoxicity compared with diclofenac [59].

Therapy with selective COX-2 inhibitory anti-inflammatory drugs promotes an imbalance in the natural prostacyclin (PGI2) and thromboxane A2 biochemical pathway [60,61]. This process is responsible for myocardial infarction, a serious adverse reaction to selective COX-2 inhibitors that caused the market withdrawal of rofecoxib from the market. Hybridization of selective COX-2 inhibitors and NO donors can increase the safety of COX-2 inhibitors by increasing vasodilation, and inhibiting the platelet aggregation and adhesion effects. In fact, several researchers have obtained different COX-2 inhibitors linked with NO donors.

**Figure 29.**
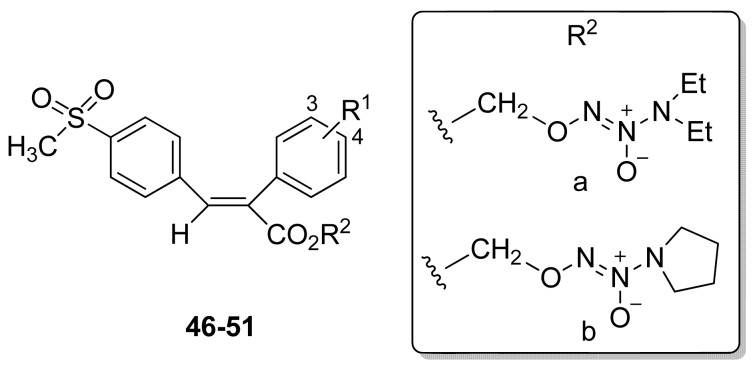
Hybrid NO-releasing/anti-inflammatory drugs (**46** R^1^ = 3-Br, R^2^ = **a; 47** R^1^ = 3-Br, R^2^ = **b; 48** R^1^ = 4-F, R^2^ = **a; 49** R^1^ = 4-F, R^2^ = **b; 50** R^1^ = 4-NHCOCH_3_, R^2^ = **a; 51** R^1^ = 4-NHCOCH_3_, R^2^ = **b**.

In 2007, Knaus and coworkers synthesized NO-releasing anti-inflammatory drug hybrids (**49-51**) possessing a 1-(*N*,*N*-diethylamino)diazen-1-ium-1,2-diolate (**a**), or 1-(pyrrolidin-1-yl)diazen-1-ium-1,2-diolate (**b**), an NO donor moiety attached via a one-carbon methylene spacer to the carboxylic acid group of (*E*)-3-(4-methanesulfonylphenyl)-2-phenylacrylic acids (Figure 29). The most potent COX-2 inhibitor compound, however, was less potent than celecoxib [62].

**Figure 30.**
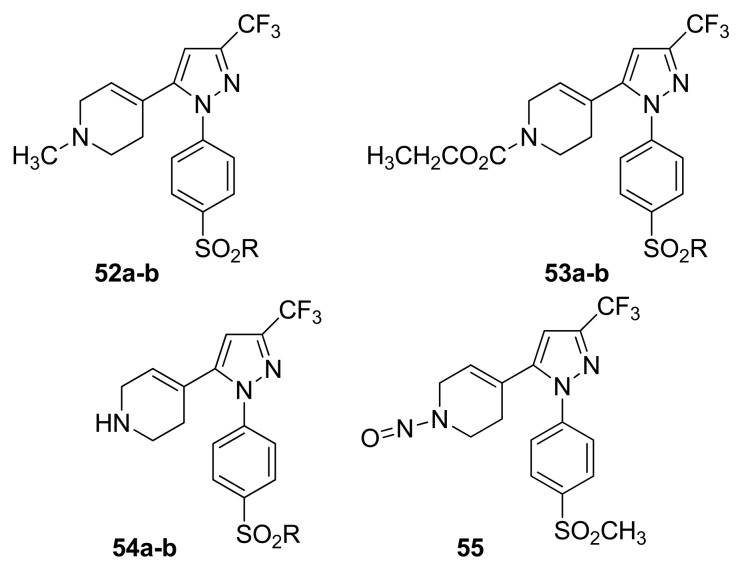
Celecoxib derivatives (**52a** R = CH_3_, 52, R = NH_2_; **53a** R = CH_3_, **53b** R = NH_2_; **54a** R = CH_3_, **54b** R = NH_2_).

Knaus and coworkers prepared a series of bioisosteres of celecoxib linked to NO donor moieties (Figure 30).

**Figure 31.**
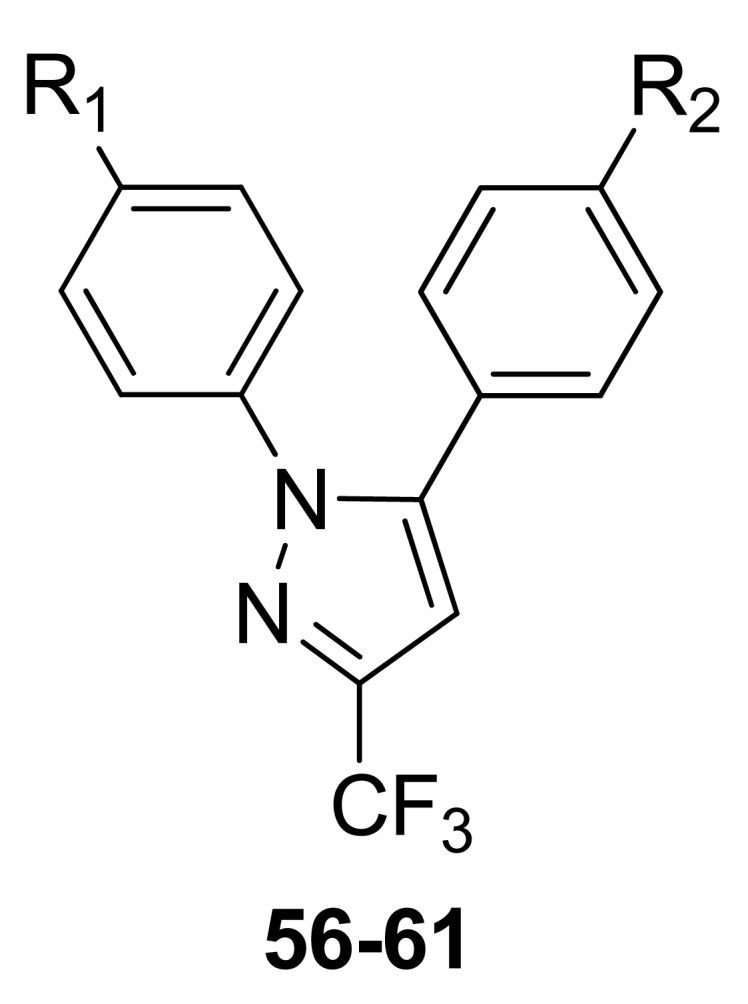
Hybrid COX2/NO donor derivative obtained by Boschi *et al*. (celecoxib R_1_ = SO_2_NH_2_, R_2_ = CH_3_; **56** R_1_ = SO_2_NH_2_, R_2_ = CH_2_OH; **57** R_1_ = SO_2_NH_2_, R_2_ = CH_2_ONO_2_; **58** R_1_ = CH_2_OH, R_2_ = CH_2_OH; **59** R_1_ = CH_2_ONO_2_, R_2_ = CH_2_ONO_2_; **60** R_1_ = CH_2_OH, R_2_ = CH_3_; **61** R_1_ = CH_2_ONO_2_, R_2_ = CH_3_).

The compounds were 4-[2-(4-methyl(amino)sulfonylphenyl)-5-trifluoromethyl-2*H*-pyrazol-3-yl]-1,2,3,6-tetrahydropyridines **52-55** possessing a variety of substituents (CH_3_, CO_2_CH_2_CH_3_, H, N = O) attached to the 1,2,3,6-tetrahydropyridyl N1-nitrogen atom. The anti-inflammatory activity of the two most potent compounds, **52a** (SO_2_CH_3_; ED_50_ = 95.3 mg/kg *p.o*) and **52b** (SO_2_NH_2_; ED_50_ = 61.2 mg/kg having an *N*-methyl-1,2,3,6-tetrahydropyridyl moiety, exhibited anti-inflammatory activities with potencies between those of the reference drugs celecoxib (ED_50_ = 10.8 mg/kg *p.o*) and aspirin (ED_50_ = 128.7 mg/kg *p.o*) [63]. In 2009 and 2010, other research groups tried to obtain a better COX-2/NO donor hybrid. Although none of the compounds demonstrated better anti-inflammatory activity than celecoxib, they showed fewer adverse effects [64,65]. The data indicated that product **57**, obtained by Boschi and coworkers, and derived from substituting the nitrooxymethyl function for the methyl group of celecoxib (IC_50_ = 1.3 ± 0.4 μM), was a weak COX-2 inhibitor, being ca. 50 times less potent than the lead drug, but it retained a good degree of COX-2 selectivity (IC_50_ = 67 ± 19 μM): it also displayed negligible COX-1 activity when tested at 100 μM concentration (Figure 31) [64].

**Figure 32.**
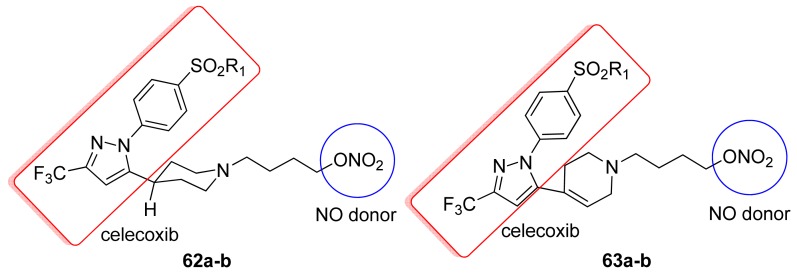
Hybrid COX-2/NO donor compounds obtained by Knaus *et al*. (**62a**, R_1_ = CH_3_; **62b**, R_1_ = NH_2_; **63a**, R_1_ = CH_3_; **63b**, R_1_ = NH_2_).

Hybrid nitric oxide donor prodrugs such as the *N*-(4-nitrooxybutyl) piperidines **62a–b** and the corresponding 1,2,3,6-tetrahydropyridine analogs **63a–b** constitute a potential class of selective COX-2 inhibitor agents that are devoid of adverse cardiovascular properties (Figure 32).

The COX-2 selectivity indices shown by compounds **62a–b** and **63a–b** are higher than those of the reference drug aspirin (selectivity index = 0.13) and comparable with those of ibuprofen (selectivity index = 2.64) [65]. The most potent COX-2 inhibitor, 1,2,3,6-tetrahydropyridyl compound **63b** having a H_2_NSO_2_ moiety, is approximately equipotent with aspirin, but less potent than celecoxib and ibuprofen [65].

## 2. Conclusions

The current treatment of inflammation is limited by several adverse effects. Combinations of adequate subunits through the molecular hybridization of anti-inflammatory drugs can create new entities with superior therapeutic activity and better safety profiles.
